# Beneficial and Detrimental Effects of Plasmin(ogen) during Infection and Sepsis in Mice

**DOI:** 10.1371/journal.pone.0024774

**Published:** 2011-09-12

**Authors:** Yongzhi Guo, Jinan Li, Elin Hagström, Tor Ny

**Affiliations:** Department of Medical Biochemistry and Biophysics, Umeå University, Umeå, Sweden; Indian Institute of Science, India

## Abstract

Plasmin has been proposed to be an important mediator during inflammation/infection. In this study, by using mice lacking genes for plasminogen, tissue-type plasminogen activator (tPA), and urokinase-type PA (uPA), we have investigated the functional roles of active plasmin in infection and sepsis. Two models were used: an infection model by intravenous injection of 1×10^7^ CFU of *S. aureus*, and a sepsis model by intravenous injection of 1.6×10^8^ CFU of *S. aureus*. We found that in the infection model, wild-type (WT) mice showed significantly higher survival rates than plasminogen-deficient (plg^-/-^) mice. However, in the sepsis model, plg^-/-^ or tPA^-/-^/uPA^-/-^ mice showed the highest survival rate whereas WT and tPA^+/-^/uPA^+/-^ mice showed the lowest survival rate, and plg^+/-^, tPA^-/-^, and uPA^-/-^ mice had an intermediate survival rate. These results indicate that the levels of active plasmin are critical in determining the survival rate in the sepsis, partly through high levels of inflammatory cytokines and enhanced STAT3 activation. We conclude that plasmin is beneficial in infection but promotes the production of inflammatory cytokines in sepsis that may cause tissue destruction, diminished neutrophil function, and an impaired capacity to kill bacteria which eventually causes death of these mice.

## Introduction

Sepsis is the culmination of complex interactions between the infecting microorganism and the host immune, inflammatory, coagulation and anti-coagulation responses [1]. Sepsis caused by infections with bacteria such as *Staphylococcus aureus* is a life-threatening condition that may lead to septic shock, resulting in multiple organ failure and death [2]. It is well known that disorders of coagulation and fibrinolysis play a major role in the development of organ dysfunction during sepsis [3]. Plasmin, a potent serine protease in fibrinolysis and the key component of the plasminogen activator (PA) system, is generated by conversion from its precursor, plasminogen, by either of two physiological PAs, tissue-type PA (tPA) and urokinase-type PA (uPA) [4]. Besides fibrinolysis, plasmin also degrades a large range of extracellular matrix substrates and activates pre-matrix metalloproteinases [5]. Plasmin has therefore been suggested to be an important upstream regulator of extracellular matrix remodeling in many tissue degradation-related innate immune processes such as cell migration, tissue remodeling, inflammation, and complement activation [6]–[8].

In addition to its roles in extracellular proteolysis, the PA system is also involved in generation of pro-inflammatory responses in the extracellular environment. Studies using plasminogen-deficient (plg^-/-^) mice have provided evidence supporting a role of the PA system in mediating the migration of inflammatory cells towards inflammatory sites [9]. *In vitro* studies have also indicated that plasmin cleaves components of the complement system, thereby releasing chemotactic complement fragments [10], [11]. Moreover, recent *in vitro* studies suggest that the PA system appears to be involved in the intracellular signaling events during inflammation. For instance, plasmin can activate the p38 mitogen-activated protein kinase (MAPK), Janus kinase (JAK), signal transducers and activators of transcription (STAT) signaling pathways in monocytes, which have been shown to be important for the inflammatory response [12]. Plasmin is also known to stimulate the release of cytokines and other inflammatory mediators by different cell types [13]. During severe infection, uncontrolled release of cytokines such as tumor necrosis factor-alpha (TNF-α) and interleukin-6 (IL-6) may cause a so-called cytokine storm [14]. An uncontrolled cytokine storm leads to sepsis, and is therefore fatal. However, although various mechanisms underlying the inflammatory response during infection have been proposed, the possible functional roles of the PA system during infection, and during sepsis especially, remain largely unknown.

In the current study, we have used single gene-deficient mice lacking plasminogen (plg^-/-^), uPA (uPA^-/-^), tPA (tPA^-/-^), and doubly deficient mice lacking both tPA and uPA (tPA^-/-^/uPA^-/-^), to study the functional roles of plasmin during *S. aureus-*induced infection and sepsis. Our data show that the plasmin shows contrasting roles in infection and sepsis.

## Results

### Reduced survival rate upon induction of infection and increased survival rate upon induction of sepsis in plg^-/-^ mice in comparison to WT mice

**Figure 1:**
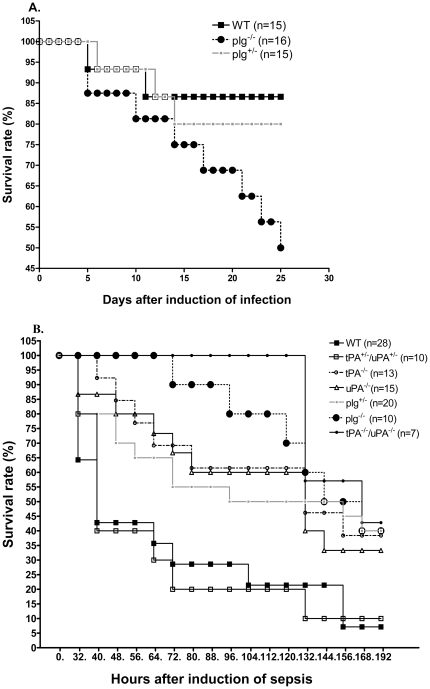
Survival curves of mice with different genotypes during infection and sepsis. **A.** Mice were inoculated with 1×10^7^ CFU of *S. aureus* to induce infection. Survival of WT (n = 15), plg^-/-^ (n = 16) and plg^+/-^ (n = 15) mice was followed for 25 days. Significant differences were found between WT and plg^-/-^ mice (log-rank test, *P*<0.05). **B.** Mice were inoculated with 1.6×10^8^ CFU of *S. aureus* to induce sepsis. Survival of WT (n = 28), tPA^+/-^/uPA^+/-^ (n = 10), tPA^-/-^ (n = 13), uPA^-/-^ (n = 15), plg^+/-^ (n = 20), plg^-/-^ (n = 10), and tPA^-/-^/uPA^-/-^ (n = 7) mice was followed for 8 days. Significant differences were found between WT mice and plg^-/-^ mice (log-rank test, *P*<0.01).

To reveal the functional roles of plasmin in infection and sepsis, we used WT, plg^+/-^, and plg^-/-^ mice, which exhibit 100%, 50%, and 0% of the normal serum plasminogen level, respectively. Infection and sepsis were achieved by intravenous injection of 1×10^7^ or 1.6×10^8^ CFU of *S. aureus,* respectively, and the survival of mice was thereafter followed for 25 days. As shown in Figure 1A, in the first 4 days after bacterial injection no mice died in the infection group. From day 5 to day 25, the survival rates of WT, plg^+/-^, and plg^-/-^ mice were 86.7%, 80%, and 50%, respectively. The survival rate in plg^-/-^ mice upon induction of infection was significantly lower (*P* = 0.04) than that in the WT mice. A contrasting result was obtained when sepsis was induced (Figure. 1B and Table 1). In this model, the median survival time in plg^-/-^ mice was 156 h, which was significantly longer (*P* = 0.009) than that in WT mice (40 h). Interestingly, the median survival time and the survival curve of plg^+/-^ mice were intermediate as compared to WT and plg^-/-^ mice. These data show that plasminogen deficiency has contrasting effects on the survival during infection and sepsis induced by *S. aureus*, and the serum levels of plasminogen affect the survival phenotype.

**Table 1:** Median survival time and mean onset time of death after induction of sepsis in different genotypes of mice.

Genotypes	Number of mice used	Median survival time (h)	Mean onset time of death (h)†
**WT**	28	40*	64.0±8.8
**tPA^+/-^/uPA^+/-^**	10	40	54.7±10.7
**tPA^-/-^**	13	132	88.5±15.9
**uPA^-/-^**	15	132	86.8±14.0
**plg^+/-^**	20	126	70.3±13.7
**plg^-/-^**	10	156	122.0±14.0
**tPA^-/-^/uPA^-/-^**	7	168	141.0±9.0

† Data presented as Mean ± SD.

,* *P<*0.05. WT group was compared with plg^-/-^ group.

### PA-deficient mice have delayed onset of death and an improved survival

Plasminogen is converted to the active enzyme plasmin by either uPA or tPA. We therefore studied *S. aureus*-induced sepsis in tPA^-/-^ or uPA^-/-^ mice, and also in doubly deficient (tPA^-/-^/uPA^-/-^) mice. As shown in Figure 1B and Table 1, the tPA^-/-^ and uPA^-/-^ mice showed delayed onset of death and significantly elevated survival rates as compared to WT mice (log-rank test *P* = 0.04). Moreover, when tPA^-/-^/uPA^-/-^ mice were used to induce sepsis, they showed the longest median survival time compared to all other genotypes. The delayed onset of death in these tPA^-/-^/uPA^-/-^ mice was even more evident than in plg^-/-^ mice (Figure 1B and Table 1). These data suggest that lack of tPA and/or uPA in mice significantly enhances the survival rate, and prolongs the onset of death in *S. aureus*-induced sepsis, thus confirming the notion that active plasmin plays a key role in the mortality of sepsis.

### Comparison of viable bacterial counts in mouse organs after induction of infection and sepsis

**Figure 2:**
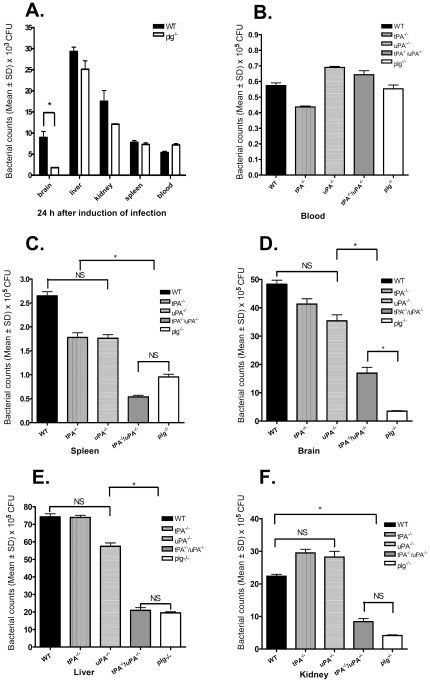
Bacterial counts in different organs and in different genotypes of mice. **A.** Bacterial counts in brain, liver, kidney, spleen, and blood of WT and plg^-/-^ mice 24 h after inoculation with 1×10^7^ CFU of *S. aureus*. **B.–F.** Bacterial counts in blood, spleen, brain, liver, and kidney 24 h after inoculation of 1.6×10^8^ CFU of *S. aureus* to different genotypes of mice. The means ± SD of 8 mice are shown. NS = not significant, * = *P*<0.05 was considered significant, by One-way ANOVA, with Bonferroni’s Multiple Comparison test.

In order to study whether there was a difference in the presence of bacteria in different organs in WT and plg^-/-^ mice after induction of infection, both genotypes of mice were inoculated with 1×10^7^ CFU of *S. aureus*, and 24 h later numbers of viable bacteria were counted from various organs. As shown in Figure 2A, the bacterial counts in liver, kidney, spleen, and blood were similar in WT and plg^-/-^ mice. The bacterial counts in brain, however, were significantly higher in WT mice than in plg^-/-^ mice. These results suggest that in the blood and in most organs, the ability to kill bacteria was similar in WT and plg^-/-^ mice at this early stage of infection. The difference in bacterial counts in the brain suggests that plasminogen may be required for bacterial invasion through the blood-brain barrier (BBB) [15], [16].

We also investigated the presence of viable bacteria in various organs after induction of sepsis in WT, tPA^-/-^, uPA^-/-^, tPA^-/-^/uPA^-/-^, and plg^-/-^ mice. As shown in Figure 2B, the numbers of viable bacteria in the blood were not significantly different in all genotypes studied at 24 h after bacterial injection. However, as shown in Figure 2C–2F, the viable bacterial counts in the spleen, brain, liver, and kidney of WT, tPA^-/-^, and uPA^-/-^ mice were significantly higher than those in tPA^-/-^/uPA^-/-^ mice and plg^-/-^ mice. The viable bacterial counts in all organs studied were not significantly different in WT, tPA^-/-^, and uPA^-/-^ mice. The bacterial counts in the brains of tPA^-/-^/uPA^-/-^ mice were significantly higher than those in plg^-/-^ mice, but this was not the case for other organs (Figure 2D). These data suggest that in sepsis, the bacterial killing ability is improved in mice with reduced levels of functional plasmin. Taken together, our data show that plasmin exhibits contrasting functions in bacterial killing in infection and sepsis.

### Higher serum levels of TNF-α, IL-6, and IL-10 in WT mice than in plg^-/-^ mice during sepsis

**Figure 3:**
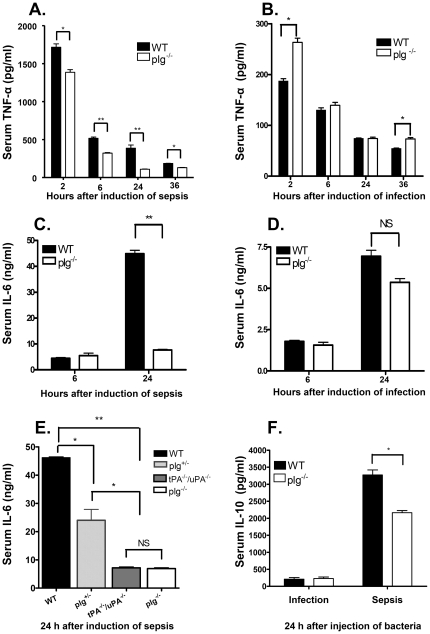
Serum levels of TNF-α, IL-6, and IL-10 during infection and sepsis. Blood samples were taken at the indicated time points. **A.** TNF-á levels in sera from WT and plg^-/-^ mice after induction of sepsis. **B.** TNF-á levels in sera from WT and plg^-/-^ mice after induction of infection.**C.** IL-6 levels in sera from WT and plg^-/-^ mice after induction of sepsis. **D.** IL-6 levels in sera from WT and plg^-/-^ mice after induction of infection. **E.** IL-6 levels in sera from WT, plg^+/-^, tPA^-/-^/uPA^-/-^, and plg^-/-^ mice after induction of sepsis. **F.** IL-10 levels in sera from WT and plg^-/-^ mice after induction of infection or sepsis. The means ± SD of 5 mice are shown. NS = not significant, * = *P*<0.05 was considered significant, and ** = *P*<0.01 was considered extremely significant, by 2-tailed *t*-test.

*In vitro* studies have indicated that plasmin stimulates the expressions of several cytokines [13]. A dramatic increase in inflammatory cytokine levels is one of the major clinical features of sepsis [17], [18]. To study how plasminogen affects the cytokine production, we studied the profiles of inflammatory cytokines in the sera of WT and plg^-/-^ mice during infection and sepsis. As shown in Figure 3A (sepsis) and 3B (infection), serum levels of TNF-α reached a maximum level at 2 h after the injection of *S. aureus*, and gradually fell thereafter in both genotypes of mice and in both infection and sepsis. After induction of sepsis (Figure 3A), significantly higher TNF-α levels were seen in WT mice than in plg^-/-^ mice at all 4 time points studied. In contrast, in the infection model (Figure 3B), the TNF-α levels were either comparable (at 6 h and 24 h) or significantly lower (at 2 h and 36 h) in WT mice than in plg^-/-^ mice.

As shown in Figure 3C and 3D, IL-6 levels were comparable in WT mice and plg^-/-^ mice at 6 h, for both the infection model and the sepsis model. However, 24 h after induction of sepsis, WT mice had 8-fold higher levels of IL-6 than plg^-/-^ mice (Figure 3C), while no difference was observed at the same time point in the infection model (Figure 3D). At 24 h after induction of sepsis, the IL-6 levels in the sera of plg^+/-^ mice were also significantly lower than those in WT mice, but significantly higher than those in plg^-/-^ and tPA^-/-^/uPA^-/-^ mice (Figure 3E).

As shown in Figure 3F, at 24 h after induction of sepsis, the IL-10 levels were significantly higher in WT mice than in plg^-/-^ mice, while the levels of IL-10 were similar between the two genotypes in the infection model. Furthermore, in both genotypes of mice, IL-10 levels were significantly higher in sepsis than in infection. Taken together, these data demonstrate that during sepsis, plg^-/-^ mice have significantly lower cytokine levels than WT mice. In addition, during sepsis, since the IL-6 levels are highest in WT mice, intermediate in plg^+/-^ mice, and lowest in both tPA^-/-^/uPA^-/-^ and plg^-/-^ mice, the increase in cytokine levels appears to be related not only to levels of plasminogen but also to levels of active plasmin.

### Improved survival in septic mice treated with IL-6 antibodies

**Figure 4:**
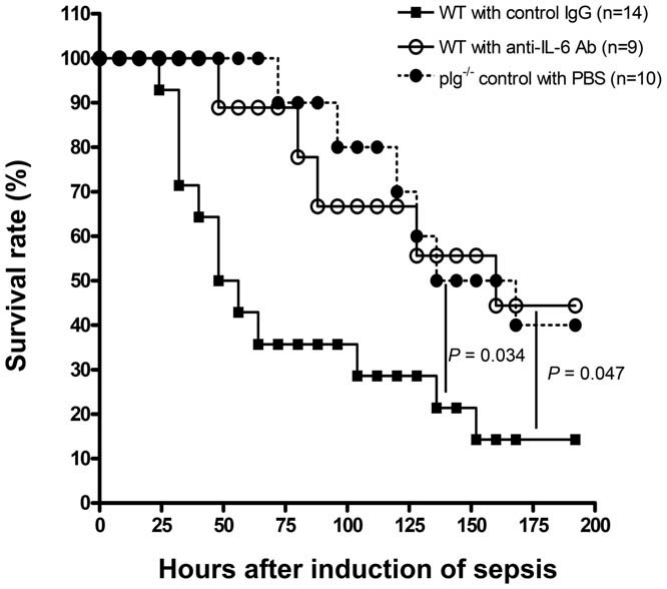
Blockade of IL-6 in WT mice during sepsis. Comparison of survival curves between anti-IL-6 antibody-treated mice (n = 9) and control IgG-treated mice (n = 14). The anti-IL-6 antibody-treated mice showed significantly improved survival as compared to control IgG-treated mice (log-rank test, *P*<0.05).

IL-6 is an important pro-inflammatory cytokine that is upregulated during sepsis [19]. As we found that the serum levels of IL-6 were significantly higher in WT mice than in plg^-/-^ mice 24 h after induction of sepsis, we tested the hypothesis that neutralization of IL-6 may delay the onset of death, or improve the survival rate in our sepsis model. As shown in Figure 4 and Table 2, when WT mice were treated with an anti-IL-6 neutralizing antibody (1.33 mg per kg body weight) at 1 h before and 24 h after the induction of sepsis, the onset of death was delayed for a period of 45 h, and the survival rate was significantly elevated as compared to mice treated with control IgG (*P*<0.05). WT mice treated with anti-IL-6 antibody showed a similar survival curve to that of plg^-/-^ mice, which suggests that lack of plasminogen improves the chances of survival from sepsis by reducing the production of IL-6.

**Table 2:** Median survival time and mean onset time of death with or without treatment of anti-IL-6 antibody (Ab) during sepsis.

Mouse group	Number of mice used	Median survival time (h)	Mean onset time of death (h)†
**WT with control IgG**	14	52*	64±12.4
**WT with anti-IL-6 Ab**	9	160	100.8±19.5
**plg^-/-^ with PBS**	10	156	120.0±13.5

† Data presented as Mean ± SD.

*, *P<*0.05. Anti-IL-6 Ab-treated group was compared to control IgG-treated group.

### Activation of STAT3 during infection and sepsis

**Figure 5:**
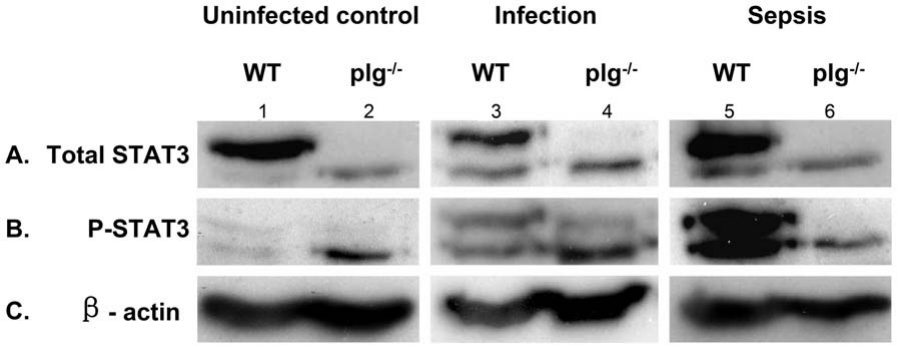
Western blot analysis of the expression levels of STAT3 in spleen neutrophils. Neutrophils from uninfected control, infection, and sepsis mice are as described in Materials and Methods. Lanes 1, 3, and 5: neutrophil lysates from WT mice; lanes 2, 4, and 6: neutrophil lysates from plg^-/-^ mice. A. Levels of total STAT3 in neutrophil lysates. B. Levels of phosphorylated STAT3 in neutrophil lysates. C. Levels of β-actin in the lysates, corresponding to each lane in A and B as internal controls. Experiments were repeated at least 3 times and representative results are shown.

STAT3 is a key molecule in mediating signaling of several inflammatory cytokines during an acute inflammatory response [20]. To study if plasminogen deficiency affect STAT3 activation, we measured the levels of phosphorylated and total STAT3 in spleen neutrophils obtained 24 h after infection and sepsis induction (Figure 5A and 5B). In the infection model, levels of STAT3 and phosphorylated STAT3 are either moderately increased (in WT mice) or remained unchanged (in plg^-/-^ mice) as compared to uninfected controls. In the sepsis model, levels of total and phosphorylated STAT3 in WT mice increased significantly upon induction of sepsis. However, the levels remained largely unchanged in plg^-/-^ mice under sepsis challenge, indicating that plasmin is involved in the activation of the STAT3 signaling pathway in sepsis, which may contribute to the differences in cytokine levels, bacterial killing ability, and mortality between WT and plg^-/-^ mice during infection and sepsis.

### Comparison of intensity of C5a on blood neutrophils in WT and plg^-/-^ mice during sepsis

**Figure 6:**
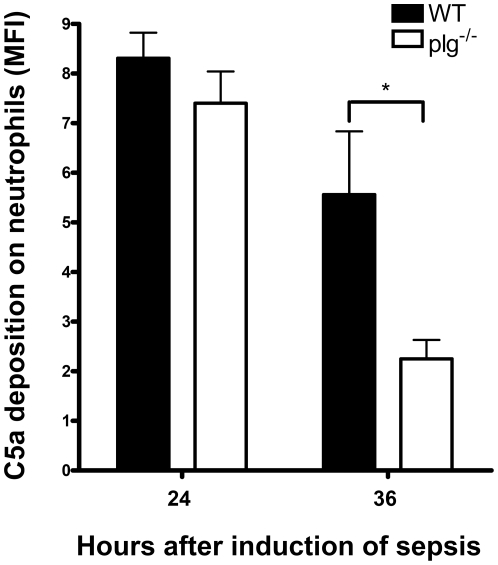
Intensity of C5a deposition on blood neutrophils in sepsis. Mice were inoculated with 1.6×10^8^ CFU of *S. aureus* to induce sepsis. Twenty-four hours and 36 h after inoculation, blood neutrophil samples were taken and stained with anti-C5a IgG. The deposition intensity is presented by mean fluorescence intensity (MFI). The means ± SD of 4 mice are shown. * = *P*<0.05 was considered significant, by2-tailed *t*-test.

Studies using a murine cecal ligation and puncture sepsis model have shown that exposure of neutrophils to C5a at concentrations occurring in human plasma during sepsis leads to neutrophil dysfunction and paralysis of signaling pathways [21]. We therefore investigated whether plasminogen deficiency may influence the surface deposition of C5a on blood neutrophils after induction of sepsis. Twenty-four and 36 h after induction of sepsis, the intensity of C5a on neutrophils was determined. As shown in Figure 6, the intensity of C5a on neutrophils was lower in plg^-/-^ mice than in WT mice at 24 h after sepsis induction and was further reduced at 36 h. The diminished deposition of C5a on neutrophils in plg^-/-^ mice during sepsis suggests that C5a may contribute to the underlying molecular mechanisms in our sepsis model.

## Discussion

In the present study, we report that plasmin plays a beneficial role in *S. aureus*-induced infection, but a detrimental role in *S. aureus*-induced sepsis. Several pro- and anti-inflammatory cytokines showed different profiles in WT and plg^-/-^ mice during infection and sepsis. In sepsis, blockade of IL-6 improved the survival of WT mice to a level that is close to plg^-/-^ mice. Furthermore, deposition of the active complement mediator C5a on the surface of neutrophils as well as activation of STAT3 is impaired in plg^-/-^ mice after induction of sepsis. These data demonstrate that plasmin has important functions in sepsis, which may go beyond its classical roles of degrading fibrin and extracellular matrix. In recent years, *in vitro* studies have suggested that plasmin plays a role in regulating signaling pathways, and in stimulating the release of cytokines and other inflammatory mediators [13]. To our knowledge, this is the first study that targets the roles of plasmin during host responses to infection and sepsis with *in vivo* models. Our results indicate that plasmin plays important roles in regulating cytokine expression, inflammatory signal transduction, bacterial killing ability, and mice survival rate during infection and sepsis.

We have previously shown that plasminogen levels are important for the incidence and severity of collagen II-induced arthritis [22]. Similarly, in the current study, our data with the sepsis model showed three types of survival phenotypes: (1) tPA^-/-^/uPA^-/-^ and plg^-/-^ mice, which cannot form active plasmin, have the highest survival rate, the longest median survival time, and the latest time of onset of death; (2) WT and tPA^+/-^/uPA^+/-^ mice have the lowest survival rate, the shortest median survival time, and the earliest time of onset of death; and (3) plg^+/-^, uPA^-/-^, and tPA^-/-^ mice have intermediate survival rate and time of onset of death. Because both tPA and uPA can activate plasminogen to functional plasmin, tPA or uPA singly deficient mice still have the capacity to convert plasminogen to active plasmin. Furthermore, tPA^-/-^/uPA^-/- ^mice have normal levels of plasminogen, but they cannot activate plasminogen to active plasmin. Thus, the survival data from the sepsis study suggest that it is the relative plasmin levels that determine the survival outcomes during sepsis.

During infection, however, WT and plg^+/-^ mice showed similar survival rates. These data indicate that unlike in sepsis, the average time of onset of death in the infection model was less dependent on the level of plasminogen. Furthermore, in sepsis, mice with normal or apparently normal ability to form plasmin (WT, tPA^-/-^, and uPA^-/-^ mice) all had significantly impaired bacterial killing ability as compared to mice that are unable to form plasmin (tPA^-/-^/uPA^-/-^ or plg^-/-^ mice). Moreover, the IL-6 levels in plg^+/-^ mice were approximately half of those in WT mice at 24 h after induction of sepsis, indicating that the relative levels of plasminogen also affect cytokine production. Based on these results, we propose that the available level of plasmin is critical for the onset of death, bacterial killing ability, and cytokine production in the development of sepsis.

STAT3 is a key molecule in mediating the signaling of many inflammatory cytokines such as TNF-α, IL-6, and IL-10 during inflammatory responses. TNF-α and IL-6 have been considered to be the primary mediators of sepsis [23], and a positive correlation has been found between serum IL-6 and TNF-α levels and multiple organ failure [24], [25]. IL-10 has a pronounced anti-inflammatory effect by reducing the level of superoxide production in neutrophils, which interferes with neutrophil-mediated cellular cytotoxicity [26]. Previous *in vitro* studies have shown that plasmin stimulates the expression of cytokines in human monocytes [13]. In our studies, we found that levels of total and phosphorylated STAT3 remained largely unchanged in plg^-/-^ mice undergoing sepsis, which is in contrast to the dramatic increased STAT3 activation in WT mice undergoing sepsis. In addition, we also found that TNF-α, IL-6, and IL-10 levels were significantly lower in plg^-/-^ mice than in WT mice during sepsis. In tPA^-/-^/uPA^-/-^ mice, the IL-6 was at a low level similar to that in plg^-/-^ mice. It is well known that the cytokine storm during sepsis induces an overwhelming inflammatory response, which leads to multiple organ failure and subsequent death. Blocking cytokines such as TNF- α, IL-6 and IL-1 did show benefit in patients with bacteremia [27]. Diminished cytokines levels in plg^-/-^ and tPA^-/-^/uPA^-/-^ mice indicate that there is a strong correlation between levels of active plasmin, cytokine expression and STAT3 activation. The data presented here suggest that reducing or removing functional plasmin in mice, leads to a higher survival rate during sepsis due to an impaired cytokine production. Studies tlb

Several studies have shown that mice with low complement activity have impaired host defense during infection [28]–[30], whereas during sepsis excessive complement activation leads to compromised innate immune functions [31]. Blockade of C5a or the C5a receptor with antibodies has been shown to greatly improve survival of rodents during sepsis [32], [33]. In the current study, the contrasting phenotypes in terms of survival rate and cytokine expression between plg^-/-^ and WT mice during infection and sepsis are in accordance with the results of previous studies in mice with dysfunction of the complement system [34]. We have also shown that both the phosphorylated and total protein expression levels of STAT3 in spleen neutrophils of plg^-/-^ mice were markedly lower than those in WT mice after induction of sepsis. Thus, one possible explanation is that plasmin is important in mediating complement activation, which subsequently activates the STAT3 signaling pathway and contributes to the different phenotypes observed in plg^-/-^ and WT mice during infection and sepsis.

In summary, the current study reveals a contrasting role of plasminogen deficiency in infection and sepsis, and suggests that pro-inflammatory plasmin plays deleterious roles during systemic inflammation (sepsis). This pro-inflammatory function of plasmin is distinctly different from its classical roles as a protease that degrades fibrin and extracellular matrix. Our findings may be potentially useful for the development of novel therapeutic strategies against infection and sepsis in humans.

## Materials and Methods

### Experimental animals

Plasminogen-heterozygous (plg**^+/-^**) mice [35] of mixed genetic background (129×C57BL/6) were intercrossed to generate wild-type (WT), plg^+/-^ and plasminogen-deficient (plg^-/-^) mice. tPA-deficient (tPA^-/-^) and uPA-deficient (uPA^-/-^) mice [36] were backcrossed 10 times to mice of C57BL/6 genetic background. The tPA/uPA doubly deficient (tPA^-/-^/uPA^-/-^) mice were generated by crossing tPA^-/-^ mice with uPA^-/-^ mice. The mice were genotyped by PCR and by measuring plasma levels of plasminogen, as described previously [37]. Male mice 8–12 weeks of age, with a body weight 22 grams or more were used for the experiments. In control experiments, the survival rates during infection and sepsis were also determined in female mice. However, no gender-related differences were found (Data not shown).The regional ethical committee of Umeå University approved all experimental protocols (Approval ID: A118-05).

### Bacterial strains

The Phillips strain of *S. aureus* isolated from a patient with osteomyelitis was kindly supplied by Dr. Höök, Gothenburg, Sweden. *S. aureus* was grown either in LB broth or on LB agar plates.

### Models for induction of infection and sepsis

#### Infection model

Mice were challenged by intravenous injection with 1×10^7^ CFU of *S. aureus* Phillips. The survival rate was examined over the whole experiment (up to 25 days).

#### Sepsis model

Mice were challenged by intravenous injection with 1.6×10^8^ CFU of *S. aureus* Phillips. The survival rate was examined over the whole experiment (up to 8 days).

### Bacterial counts in blood and different organs

Twenty-four hours after injection of the bacteria in amounts compatible with either infection or sepsis, the mice were killed and the blood, brain, spleen, liver, and kidney were collected and homogenized in 2 ml sterile PBS. After serial dilution, the homogenates and the blood were spread on LB agar plates and incubated at 37°C for 24 h. Colonies were counted and expressed as CFU/organ.

### ELISA

At the indicated time points after induction of infection or sepsis, approximately 600 µl blood were collected, placed on ice and allowed to clot before centrifugation at 3000×*g* for 10 min. Serum samples were then used for ELISA. The serum levels of TNF-α, IL-6, and IL-10 were measured using ELISA kits according to the instructions of the manufacturer (Nordic Biosite, Täby, Sweden).

### Western blot analysis

Twenty-four hours after induction of infection or sepsis, spleens were collected and homogenized on ice, and the neutrophils were isolated by Percoll gradients (P4937; Sigma-Aldrich, Stockholm, Sweden). Neutrophils were lysed with 1% NP-40 and protease inhibitor cocktail. Thereafter, protein concentrations were measured by the BCA protein assay method (Pierce Biotechnology, Rockford, IL). Thirty micrograms of protein was electrophoresed by SDS-PAGE (7.5%), and transferred to polyvinylidene difluoride membranes (Amersham, UK) for western blot. Anti-STAT3 and anti-phosphorylated-STAT3 (Tyr^705^) antibodies were obtained from Cell Signaling Technology (Boston, MA). The mouse monoclonal antibodies to β-actin were purchased from Sigma-Aldrich, Sweden. Western blot analyses were carried out as described by Persau et al [38].

### Detection of C5a on blood neutrophils during sepsis by flow cytometry

Flow cytometric staining was performed on whole blood from 5 WT mice and 5 plg^-/-^ mice at 24 and 36 h, respectively, after sepsis induction. Leukoctes isolation was essentially followed by Zhang et al [39]. Cells were incubated with rat antibody to mouse neutrophils (MCA771G; Serotec, UK) and goat antibody to mouse C5a (AF2150; Minneapolis, MN) for 30 min on ice. After washing the secondary antibodies with FACS buffer, FITC-labeled sheep anti-rat IgG diluted 1∶100 (AAR10F; Serotec, UK) or PE-labeled donkey anti-goat IgG diluted 1∶200 (Rockland Immunochemicals, PA) was added. The cells were washed once with FACS buffer and resuspended in 500 µl PBS. The samples were analyzed by double-colour fluorescence flow cytometry with cytomics FC500 (Beckman Coulter) for the detection of C5a intensity on blood neutrophils.

### IL-6 blockade in WT mice 1 h prior to and 24 h after bacterial challenge

A rat monoclonal antibody (anti-IL-6; BD PharMingen, San Diego, CA) that specifically neutralize recombinant mouse IL-6 were used. The antibody were given to 9 WT mice at a dose of 1.33 mg per kg body weight at 1 h prior to and 24 h after the induction of sepsis. As controls, 14 WT mice were given control IgG (R3-34; BD PharMingen, San Diego, CA) at the same dosage. The mice were monitored every 8 h, and were killed 192 h after induction of sepsis.

### Statistical analysis

Results of bacterial counts are expressed as the mean ± SD. Data sets were analyzed using One-way ANOVA, with Bonferroni’s Multiple Comparison test. Results of cytokine levels are expressed as the mean ± SD. Data sets were analyzed using 2-tailed *t*-test. To compare the survival curves among different genotypes of mice, log-rank test was used. *P* values less than 0.05 were considered significant.
